# *Clostridium butyricum* Protects IPEC-J2 Cells from ETEC K88-Induced Oxidative Damage by Activating the Nrf2/ARE Signaling Pathway

**DOI:** 10.1155/2021/4464002

**Published:** 2021-07-21

**Authors:** Caixia Dou, Zhiyuan Shang, Jiayun Qiao, Yimeng Wang, Haihua Li

**Affiliations:** ^1^College of Life Sciences, Tianjin Key Laboratory of Animal and Plant Resistance, Tianjin Normal University, Tianjin 300387, China; ^2^Tianjin Key Laboratory of Agricultural Animal Breeding and Healthy Husbandry, College of Animal Science and Veterinary Medicine, Tianjin Agricultural University, Tianjin 300384, China

## Abstract

*Clostridium butyricum* (CB) is a naturally occurring probiotic compound that can alleviate the oxidative damage induced by enterotoxigenic *Escherichia coli* K88 (ETEC K88) in porcine intestinal epithelial (IPEC-J2) cells. In this study, we investigate the molecular mechanism underlying this effect. Based on cell viability, malondialdehyde (MDA), superoxide dismutase (SOD), glutathione peroxidase (GPX) assessments, the optimal concentration of ETEC K88 was determined to be 1 × 10^3^ cfu/mL. Viable bacteria counts in cells pretreated with CB and then infected with ETEC K88 show that CB can adhere to IPEC-J2 cells and that optimal adhesion is achieved at the multiple infection index (MOI) of 50 at 3 h of pretreatment. The results of qPCR indicate that although ETEC significantly decreases the expression levels of antioxidant enzymes regulated by NF-E2-related factor 2 (Nrf2) compared to the control group, CB reverses this effect. To confirm that Nrf2 is directly involved in the mechanism by which CB alleviates oxidative stress, siRNA was used to silence the expression of *Nrf2* gene in IPEC-J2 cells. Compared to the NC+ETEC and siRNA+ETEC groups, the expressions of *SOD1*, *SOD2*, *GPX1*, and *GPX2* in the NC+CB+ETEC and siRNA+CB+ETEC groups are significantly increased at 12 h and 24 h. This shows that CB can reduce ETEC K88-induced oxidative damage in IPEC-J2 cells by activating the expression of antioxidant enzymes implicated in the Kelch-like ECH-associated protein-1- (Keap1-) Nrf2/antioxidant response element (ARE) signaling pathway.

## 1. Introduction

Oxidative stress, a condition induced by the imbalance between the oxidant and antioxidant systems in cells and tissues, destroys intestinal homeostasis by stimulating the production of a large number of reactive oxygen species (ROS), which affects the stability of nucleic acids, proteins, and lipids and increases cell apoptosis, leading to intestinal mucosal injury [1, 2]. Enterotoxigenic *Escherichia coli* (ETEC) is a pathogenic bacteria that causes diarrhea and intestinal disease in weaned piglets. It combines with the microvilli of intestinal epithelial cells and with receptors on the surface of cells through various types of fimbriae adhesins; then, it propagates in large numbers, which releases enterotoxin, a protein that induces diarrhea [3]. Studies conducted on piglet intestinal epithelial cells have shown that ETEC K88-activated oxidative stress destroys the intestinal epithelial barrier and increases permeability, which eventually leads to diarrhea [4]. In addition to piglets, ETEC K88 can activate oxidative stress in mice [5, 6].

*Clostridium butyricum* (CB), a naturally existing probiotics in animal and human intestines, produces butyric acid, regulates intestinal pH, maintains a healthy intestinal environment, and protects intestines from pathogenic bacteria [7]. Diets supplemented with CB can reduce ETEC K88-induced inflammatory response and intestinal epithelial cell damage in weaned piglets [8]. Moreover, these diets improve the intestinal barrier function and digestive enzyme activity in broilers infected with ETEC K88 [9]. According to previous studies, CB promotes the expression of NF-E2-related factor 2 (*Nrf2*) in mice and alleviates oxidative stress [10–12]. Therefore, the anti-inflammatory, antioxidant, and immune-enhancing effects of this probiotic may be related to the Kelch-like ECH-associated protein-1- (Keap1-) Nrf2/antioxidant response element (ARE) signaling pathway, which is one of the most important antioxidative stress pathways in cells. Under the condition of normal physiological, Nrf2 is located in the cytoplasm and relies on ubiquitination degradation to maintain it in a stable state of low inactivity [13]. Under the condition of oxidative stress, Nrf2 accumulates in the nucleus where it binds to ARE, thereby initiating the expression of a series of downstream protective genes, such as glutathione peroxidase (*GPX*) and superoxide dismutase (*SOD*) [14]. SOD converts superoxide radicals from extracellular stimuli (including ionizing radiation and oxidative damage) and those generated within the mitochondrial matrix via the electron transport chain as byproducts of oxygen metabolism to hydrogen peroxide, for which GPX is the primary hydrogen peroxide scavenging enzyme, and subsequently converts hydrogen peroxide to water [15–17]. In this study, we explore the molecular mechanism by which CB alleviates ETEC K88-induced oxidative stress in IPEC-J2 cells. In particular, we assess whether the effect of the probiotic is related to the Nrf2/ARE signaling pathway.

## 2. Materials and Methods

### 2.1. Experimental Strains and Reagents

ETEC K88 was preserved in the laboratory of the School of Animal Science and Veterinary Medicine at Tianjin Agricultural University. CB was isolated from the feces of healthy piglets.

RPMI 1640 basic culture medium, fetal bovine serum (FBS), and trypsin EDTA digestion solution were purchased from Gibco (New York, USA). PBS buffer, penicillin, Triton X-100, and dimethyl sulfoxide (DMSO) were purchased from Solarbio (Beijing, China). Lipofectamine 2000 transfection kit and Opti-MEM were supplied by Invitrogen (Carlsbad, USA), whereas the cell counting kit-8 (CCK-8) was obtained from Dojindo (Kumamoto, Japan). The MDA, GPX, and SOD enzyme-linked immunoassay (ELISA) kit was purchased from Nanjing Jiancheng Bioengineering Institute (Nanjing China). The reverse transcription kit and real-time PCR kit were bought from GeneCopoeia (Rockville, USA). Finally, the primary antibodies of Nrf2 (ab92946) and GAPDH (mAbcam 9484) and the secondary antibodies HRP Goat anti-rabbit IgG (ab205718) and HRP Goat anti-mouse IgG (ab205719) were purchased from Abcam (Cambridge, UK).

### 2.2. Cell Culture

The IPEC-J2 cell line was obtained from Shanghai Guandao Biological Engineering Co., Ltd. The cells were cultured in a medium containing 89% RPMI 1640 basic medium, 10% FBS, and 1% penicillin at 37°C and 5% CO_2_.

### 2.3. Cell Viability Assay

The cell suspension was seeded in 96-well plates with 1 × 10^4^ cells per well (100 *μ*L in each well). After 24 h, ETEC K88 was added to the cells at the concentrations of 0 (blank), 1 × 10^1^, 1 × 10^2^, and 1 × 10^3^, and 5 × 10^3^ and 1 × 10^4^ cfu/mL, with three repetitions in each group. The mixtures were incubated for 3 h, 6 h, 12 h, 18 h, and 24 h, and then, the cells were washed three times with PBS in order to move the adherent ETEC K88. Subsequently, 10 *μ*L CCK-8 reagent added to each well, followed by incubation for another 3 h. Finally, the absorbance of the samples was at 450 nm using a microplate reader (TECAN, Mannendorf, Switzerland).

### 2.4. Determination of GPX, SOD, and MDA Levels

After seeding in a 6-well plate (1 × 10^6^ cells per well) for 24 h. IPEC-J2 cells were treated with different concentrations of ETEC K88 (0, 1 × 10^1^, 1 × 10^2^, and 1 × 10^3^ cfu/mL) for 3 h, 6 h, 12 h, 18 h, and 24 h, with three repetitions in each group. The cell culture supernatant was collected and the content of malondialdehyde (MDA) and the activity of SOD and GPX were detected using the ELISA kit, according to the manufacturer's instructions.

### 2.5. Cell Adhesion Ability of CB

To study the time dependence of CB cell adhesion ability, IPEC-J2 cells were first seeded in a 24-well plate at the concentration of 1 × 10^5^ cells per well. After 24 h, CB was added to the cells at 50 multiplicity of infection (MOI = number of bacteria/number of cells), and the mixtures were incubated for 3 h, 6 h, 12 h, and 24 h, with three repetitions in each group. The dose dependence of cell adhesion of CB was incubating the cultured cell with the probiotic at 1, 10, 50 and 100 MOI for 3 h. After cleaning three times with PBS to remove, the nonadherent CB, 100 *μ*L 0.5% Triton X-100 were added to each well, followed by incubation for 8 min. Subsequently, the cells were lysed, and then, 900 *μ*L PBS was added to terminate lysis. Finally, the samples were diluted by varying degrees, and the number of adherent CB was determined.

### 2.6. RNA Extraction and Quantitative Real-Time PCR Analysis

After 24 hours of culture in a 6-well plate, IPEC-J2 cells were pretreated with CB (MOI = 50) for 3 h, washed with PBS, and then treated with ETEC K88 (1 × 10^3^ cfu/mL) for 3 h, 6 h, 12 h, and 24 h. Subsequently, the RNA in the cells was extracted using the nucleic acid extraction kit (OD260/OD280 ratio of 1.8-2.0); then, it was reverse transcribed according to the instructions of the RT kit, using cDNA as a template for quantitative analysis. The total volume of the reaction solution was 20 *μ*L, which includes 10 *μ*L PCR mix, 3 *μ*L template, 3 *μ*L water, and 4 *μ*L primer. The relative mRNA expression of the target gene was calculated by 2^-△△Ct^.

The primer sequences are listed in Table 1. The primers of *SOD1* [18], *SOD2* [19], *GPX1* [20], *Nrf2* [21], and glyceraldehyde-3-phosphate dehydrogenase (*GAPDH*) [22] were designed according the method described in previous reports, and the primers of *GPX2* were designed with primer 5. All primer sequences were synthesized by Sangon Biotech Co., Ltd. (Shanghai, China).

### 2.7. Western Blot Analysis

IPEC-J2 cells were seeded in a 6-well plate, pretreated with CB for 3 h, and then treated with 1 × 10^3^ cfu/mL ETEC K88 for 12 h. Afterward, the cells were lysed on ice for 30 min using the NP-40 lysis buffer with 0.1% PMSF; then, they were centrifuged at 4°C and 12 000 r/min for 10 min. The proteins were separated on a 12% SDS/PAGE gel, semidried for 40 min, and then transferred to a polyvinylidene fluoride (PVDF) membrane. After blocking with skimmed milk powder for 2 h, the membrane was incubated overnight with Nrf2 and GAPDH primary antibodies at 4°C, followed by cleaning with TBST and incubation with the secondary antibodies at room temperature for 2 h. The imprint was observed and recorded using the ECL chemiluminescence kit, and the concentration of each band was analyzed by the ImageJ analysis software.

### 2.8. siRNA Transfection in IPEC-J2 Cells

The forward (5′-gccaugaugucugutt-3′) and reverse (5′-aucagaugaugauggctt-3′) Nrf2 interference sequences were synthesized by Sangon Biotech Co., Ltd. (Shanghai). Solutions of 10 *μ*L Lipofectamine 2000 in 500 *μ*L Opti-MEM medium and 10 *μ*L siRNA or negative control (NC) (0.4 *μ*mol/L) in 500 *μ*L Opti-MEM medium were separately incubated in RNase-free centrifuge tubes at room temperature for 5 min, and then, they were mixed and incubated together for another 20 min. IPEC-J2 cells seeded in a 6-well plate were with siRNA or NC for 6 h and then cultured in complete medium for 18 h. After pretreatment with CB for 3 h, the cells were treated with 1 × 10^3^ cfu/mL ETEC K88 for 3 h, 6 h, 12 h, and 24 h, with three repetitions in each group. The experiment was divided into seven groups: control, NC, siRNA, NC+ETEC, siRNA+ETEC, NC+CB+ETEC, and siRNA+CB+ETEC, with three replications in each group.

### 2.9. Statistical Analysis

SPSS 20.0 (SPSS Inc., Chicago, USA) and least significant difference (LSD) were used for one-way ANOVA statistical analysis and multiple comparisons, respectively. The column chart constructed using the GraphPad Prism 5.0 (Graphpad Prism 5.0 Software Inc., Califomia). *P* values < 0.05 were considered to be statistically significant.

## 3. Results

### 3.1. Viability of IPEC-J2 Cells Treated with ETEC K88

**Figure 1:**
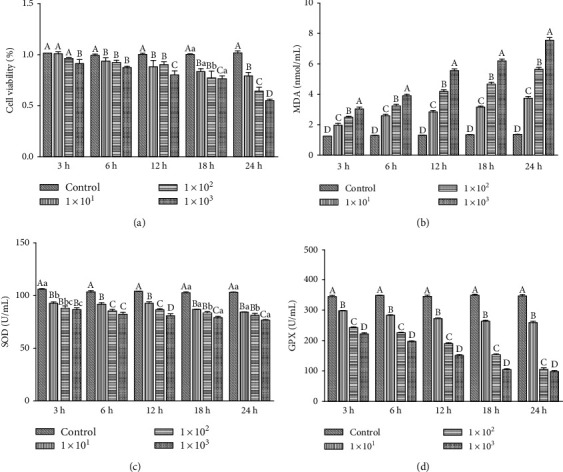
Viability of IPEC-J2 cells treated with ETEC K88. (a) After 3 h, 6 h, 12 h,18 h, and 24 h of treatment with 1 × 10^1^, 1 × 10^2^, and 1 × 10^3^ cfu/mL ETEC K88 the viability of cells was determined via CCK-8. (b–d) The levels of MDA, SOD, and GPX in the supernatant of cells treated with ETEC K88 for 3 h, 6 h, 12 h, 18 h, and 24 h were detected by ELISA. The results were mean ± SEM of three independent preparations. Value columns with different lowercase letters mean *P* < 0.05, while with different capital letters mean *P* < 0.01.

The viability of IPEC-J2 cells treated with varying concentrations of ETEC K88 for different time durations was assessed using the CCK-8 and ELISA kits. The obtained results (Figure 1(a)) show that, after 3 h of treatment with 1 × 10^1^ and 1 × 10^2^ cfu/mL of ETEC K88, the viability of cells does not change significantly (*P* > 0.05 compared to the control group). However, the cells incubated with 1 × 10^3^ cfu/mL ETEC K88 for 3 h are appreciably less viable than those in the control group (*P* > 0.05). When the treatment is prolonged to 6 h, a significant decreased in cell viability is observed, irrespective of the concentration of ETEC K88 (1 × 10^1^, 1 × 10^2^, or 1 × 10^3^ cfu/mL) (*P* < 0.01) compared to the control group. As the treatment duration increases beyond 6 h (12 h, 18 h, and 24 h), the cell viability decreases further. Notably, a greater decrease is detected at 1 × 10^3^ cfu/mL than at 1 × 10^1^ or 1 × 10^2^ cfu/mL, and the difference between the two sets of concentration is significant (*P* < 0.05 for 12 h and 18 h treatment; *P* < 0.01 for 24 h treatment). Moreover, the MDA content increases significantly with increasing concentration of ETEC K88 (*P* < 0.01), while the activities of SOD and GPX decrease significantly (*P* < 0.01). For same time treatments, the highest MDA content and the lowest SOD and GPX contents are observed for cells treated with 1 × 10^3^ cfu/mL ETEC K88 (Figures 1(b)–1(d)). Meanwhile, for same dose treatments, greater MDA content and smaller SOD and GPX contents are detected at longer incubation times. It should be noted that the cell culture media containing of 5 × 10^3^ and 1 × 10^4^ cfu/mL ETEC K88 were found to be turbid and that most cells in these media died after 24 h. Based on the obtained results, the ETEC K88 concentration of 1 × 10^3^ cfu/mL was selected for subsequent experiments.

### 3.2. Cell Adhesion Ability of CB

**Figure 2:**
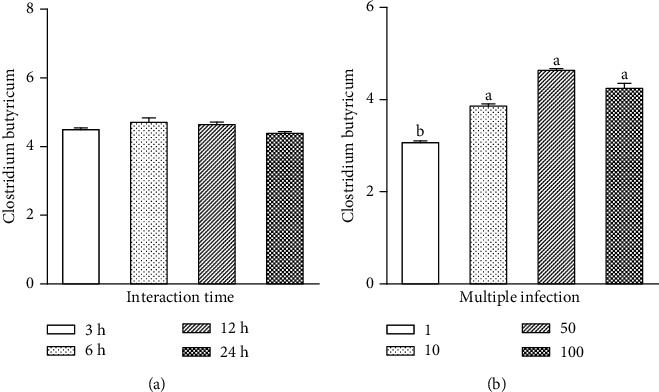
Cell adhesion ability of CB. (a) CB was added to the cells at 50 multiplicity of infection and the mixtures were incubated for 3 h, 6 h, 12 h, and 24 h. (b) CB with MOI of 1, 10, 50, and 100 was added to incubate the cells for 3 h. The results were mean ± SEM of three independent preparations. Value columns with different lowercase letters mean *P* < 0.05.

Figure 2 presents the concentration and time dependence of CB cell adhesion ability. As shown in Figure 2(a), the adhesion index of cells increases within the first 12 h of incubation with the probiotic; then, it decreases after treatment for 24 h. However, the difference between the indexes calculated at different incubation times is not significant (*P* > 0.5). Although the adhesion index does not appreciably depend on time, it varies significantly with concentration. Specifically, the cell adhesion ability of CB increases with increasing MOI in the range of 1-50 and then decreases at MOI 100. Based on microscopic observation, the culture medium containing CB at MOI 100 exhibits some cells dead and floating cells. This indicates that at high concentrations (MOI ≥ 100), the probiotic damages IPEC-J2 cells (Figure 2(b)). Based on these results, subsequent experiments were conducted under the conditions of 3 h CB pretreatment at 50 MOI.

### 3.3. Effect of CB on Nrf2 Signaling in ETEC K88-Infected IPEC-J2 Cells

**Figure 3:**
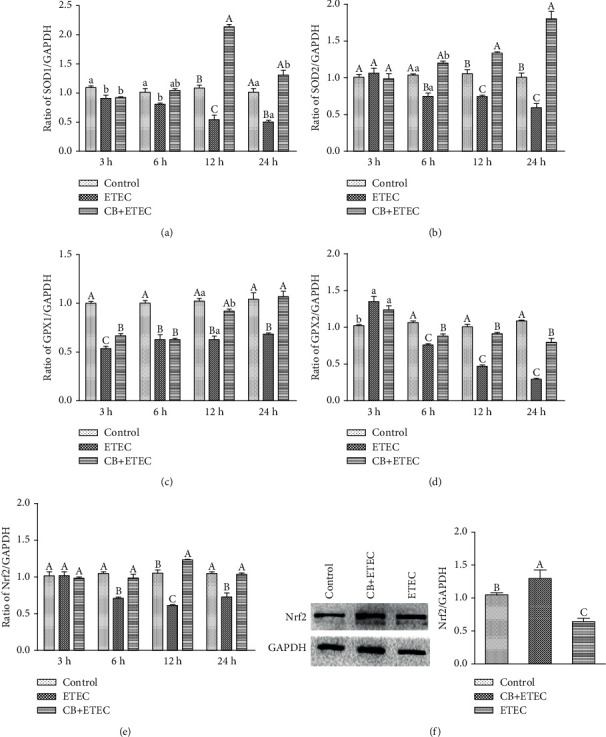
Effect of CB on Nrf2 signaling in ETEC K88-infected IPEC-J2 cells. (a–e) Real-time PCR was used to detect the relative expression level of *SOD1*, *SOD2*, *GPX1*, *GPX2*, and *Nrf2*, calculated against the housekeeping gene *GAPDH*. (f) The Nrf2 protein levels after ETEC infected were measured by Western blot analysis. The results were mean ± SEM of three independent preparations. Value columns with different lowercase letters mean *P* < 0.05, while with different capital letters mean *P* < 0.01.

Compared to the control group, the expression levels of *SOD1* and *GPX1* in ETEC-infected cells are significantly lower (*P* < 0.05) (Figures 3(a) and 3(c)). Similarly, ETEC infection decreases the expression levels of *SOD2* and *Nrf2* after treatment for more than 3 h (*P* < 0.01) (Figures 3(b) and 3(e)). As for *GPX2*, its expression level increases significantly after 3 h of treatment with ETEC and then decreases at 6 h, 12 h, and 24 h (*P* < 0.01) (Figure 3(d)). The *SOD1* expression level detected for the CB+ETEC group at 3 h and 6 h is similar to that of the ETEC group (*P* > 0.05); however, it becomes significantly greater after 12 h and 24 h of treatment (*P* < 0.01). At 3 h, the expression of *SOD2* in the CB+ETEC group does not change appreciably compared to the ETEC group, but it increases significantly at 6 h, 12 h, and 24 h (*P* < 0.01). *GPX1* and *Nrf2* expressions do not exhibit significant change at 6 h and 3 h, respectively (*P* < 0.01); however, the expression levels of *Nrf2* and its downstream genes in the CB+ETEC group increase at 12 h. This indicates that after 12 h of treatment, the level of *Nrf2* protein is significantly increased in the CB+ETEC group (*P* < 0.01) and significantly decreased in the ETEC group (*P* < 0.01), compared to the control group. As shown in Figure 3(f), the change in Nrf2 protein level is consistent with the profile of gene expression. These results indicate that ETEC can induce oxidative stress injury and reduce the expression of antioxidant genes in cells. Pretreatment with CB alleviates the damaging effects of ETEC.

### 3.4. Effect of CB on ETEC K88-Induced Damage in IPEC-J2 Cells

**Figure 4:**
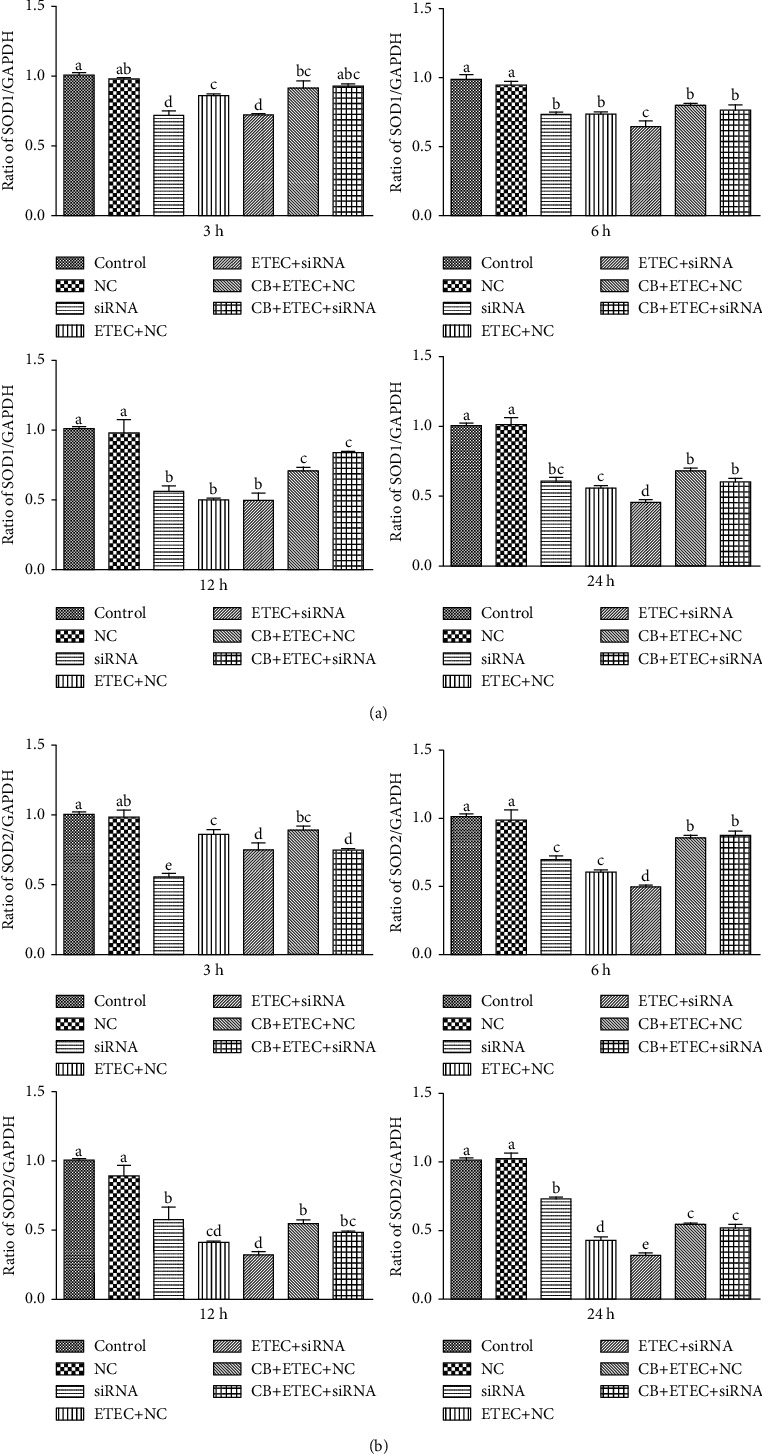
Role of SOD on ETEC K88-induced damage promoted by CB. (a, b) IPEC-J2 cells incubated with Nrf2-specific siRNA (siRNA) and Nrf2 nonspecific siRNA control (NC) for 6 h. After pretreatment with CB for 3 h, the cells were treated with 1 × 10^3^ cfu/mL ETEC K88 for 3 h, 6 h, 12 h, and 24 h. The contents of *SOD1* and *SOD2* were determined by qPCR. The results were mean ± SEM of three independent preparations. Value columns with different lowercase letters mean *P* < 0.05.

As shown in Figure 4, the expressions of *SOD1* and *SOD2* in the NC group are similar to those detected in the control group (*P* > 0.05). In the siRNA, NC+ETEC, and siRNA+ETEC groups, the expression levels of *SOD1* and *SOD2* are significantly lower than those corresponding to the control group at all times (*P* < 0.05). Compared to the NC+ETEC group, the NC+CB+ETEC group exhibits significantly increased *SOD1* expression at 12 h and 24 h (*P* < 0.05) with no appreciable change at 3 h and 6 h (*P* > 0.05) and significantly increased *SOD2* expressions at 6 h, 12 h, and 24 h (*P* < 0.05) with no appreciable change at 3 h (*P* > 0.05). Compared to the siRNA+ETEC group, *SOD1* expressions in the siRNA+CB+ETEC group are significantly increased at all times (*P* < 0.05), whereas *SOD2* expressions are increased only at 6 h, 12 h, and 24 h (*P* < 0.05). The results showed that the expression of SOD tended to decrease after the downregulation of *Nrf2* expression, and the addition of CB could upregulate the decrease of *SOD* expression caused by ETEC.

**Figure 5:**
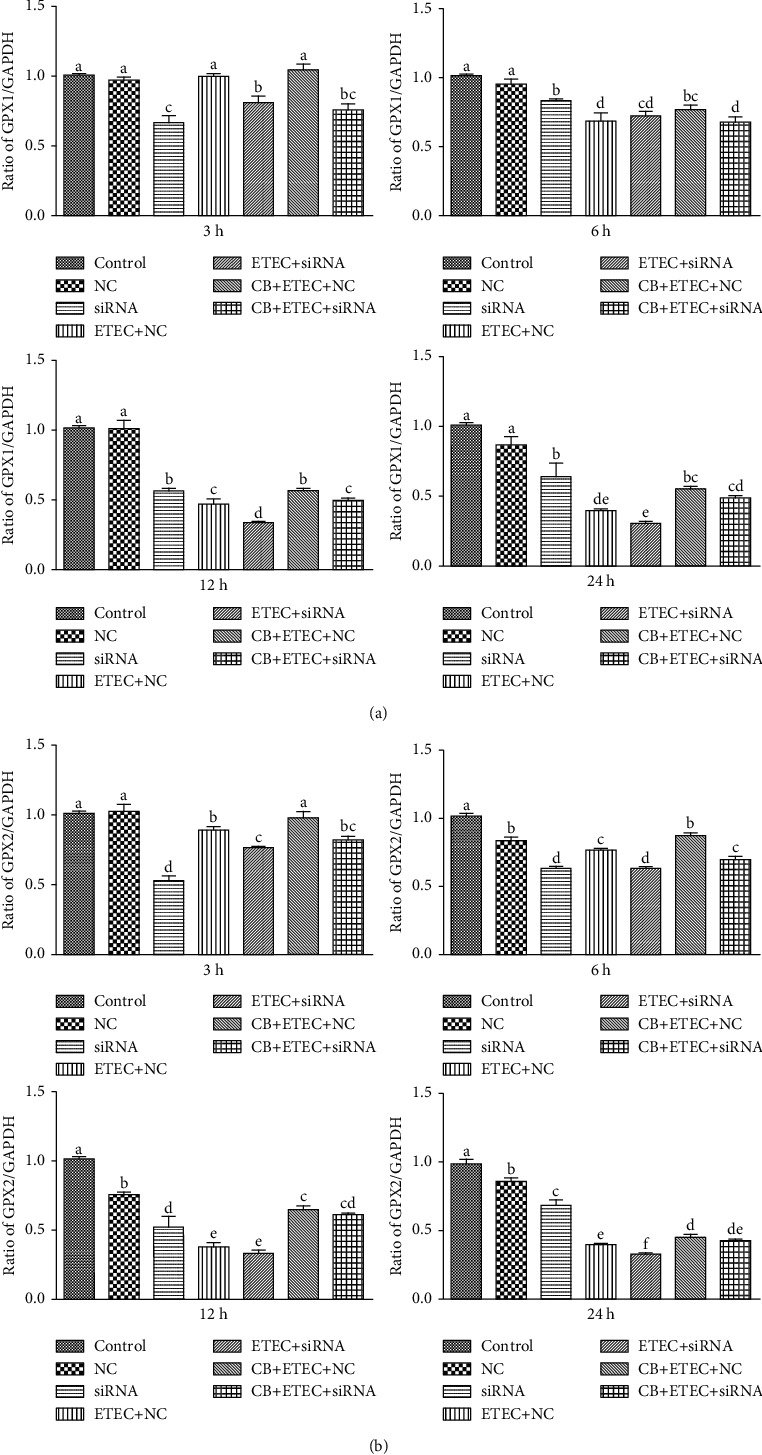
Role of GPX on ETEC K88-induced damage promoted by CB. (a, b) IPEC-J2 cells incubated with Nrf2-specific siRNA (siRNA) and Nrf2 nonspecific siRNA control (NC) for 6 h. After pretreatment with CB for 3 h, the cells were treated with 1 × 10^3^ cfu/mL ETEC K88 for 3 h, 6 h, 12 h, and 24 h. The contents of *GSH1* and *GSH2* were determined by qPCR. The results were mean ± SEM of three independent preparations. Value columns with different lowercase letters mean *P* < 0.05, while with different capital letters mean *P* < 0.01.

As shown in Figure 5, the expressions of *GPX1* in the NC group are similar to those detected in the control group (*P* > 0.05); however, the expression of *GPX2* is significantly reduced except at 3 h (*P* < 0.05). In the siRNA, NC+ETEC, and siRNA+ETEC groups, the expression levels of *GPX1* and *GPX2* are significantly lower than those corresponding to the control group at 6 h, 12 h, and 24 h (*P* < 0.05). Compared to the NC+ETEC group, the NC+CB+ETEC group exhibits significantly increased *GPX1* expressions at 6 h, 12 h, and 24 h (*P* < 0.05) with no appreciable change at 3 h (*P* > 0.05) and significantly increased *GPX2* at all times (*P* < 0.05). Compared to the siRNA+ETEC group, *GPX2* expressions are increased only at 6 h, 12 h, and 24 h (*P* < 0.05). The expressions of *GPX1* are increased at 12 h and 24 h (*P* < 0.05). The results showed that the expression of *GPX* tended to decrease after the downregulation of *Nrf2* expression, the addition of CB could upregulate the decrease of *GPX* expression caused by ETEC, and treating the cells with siRNA restricts the probiotic effect of CB.

**Figure 6:**
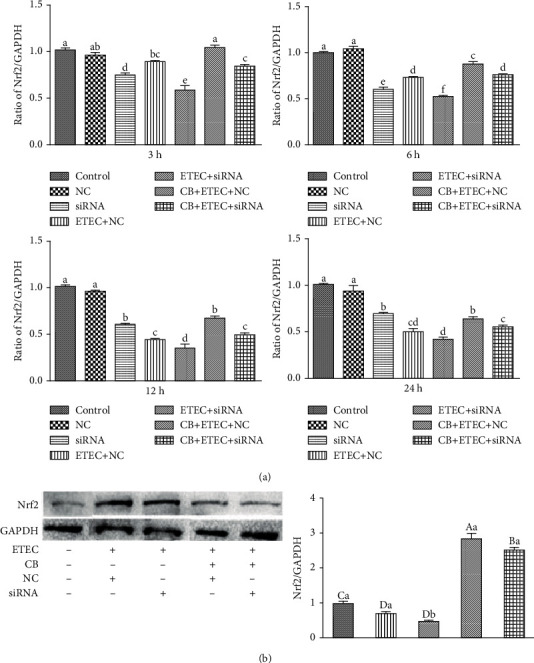
Role of Nrf2 on ETEC K88-induced damage promoted by CB. (a) IPEC-J2 cells incubated with Nrf2-specific siRNA (siRNA) and Nrf2 nonspecific siRNA control (NC) for 6 h. After pretreatment with CB for 3 h, the cells were treated with 1 × 10^3^ cfu/mL ETEC K88 for 3 h, 6 h, 12 h, and 24 h. The contents of *Nrf2* were determined by qPCR. (b) After pretreatment with CB for 3 h, the cells were treated with 10^3^ cfu/mL ETEC K88 for 12 h. Determination of Nrf2 proteins content by Western blot. The results were mean ± SEM of three independent preparations. Value columns with different lowercase letters mean *P* < 0.05, while with different capital letters mean *P* < 0.01.

As shown in Figure 6, the expression of *Nrf2* in the NC group is similar to those detected in the control group (*P* > 0.05). In the siRNA, NC+ETEC, and siRNA+ETEC groups, the expression level of *Nrf2* is significantly lower than those corresponding to the control group at all times (*P* < 0.05). Compared to the NC+ETEC group, the NC+CB+ETEC group exhibits significantly increased *Nrf2* at all times (*P* < 0.05). This indicates that CB can enhance the protective effect of Nrf2. Compared to the siRNA+ETEC group, *Nrf2* expressions in the siRNA+CB+ETEC group are significantly increased at all times (*P* < 0.05) (Figure 6(a)), indicating that CB can increase the expression of *Nrf2*.

The expression of *Nrf2* and its downstream genes in the siRNA+CB+ETEC and NC+CB+ETEC groups increase significantly at 12 h, resulting in changed Nrf2 protein expression. Indeed, the levels of Nrf2 protein in the NC+ETEC and siRNA+ETEC groups are substantially lower than that detected in the control group (*P* < 0.01). Moreover, the siRNA+ETEC group exhibits reduced Nrf2 protein expression compared to the NC+ETEC group (*P* < 0.05), whereas the expression of this protein in the NC+CB+ETEC group is increased (*P* < 0.01). Notably, Nrf2 protein expression in the siRNA+CB+ETEC group is greater than that in the siRNA+ETEC group (*P* < 0.01), but less protein is detected in the siRNA+CB+ETEC group than that in the NC+CB+ETEC group (*P* < 0.01) (Figure 6(b)). These results indicated that the downregulation of Nrf2 signaling alleviates this damage by upregulating the Nrf2 signaling pathway. Moreover, treatment with siRNA restricts the probiotic effect of CB on IPEC-J2 cells.

## 4. Discussion

In biological systems, ROS are mainly produced via metabolic reactions occurring in the mitochondria, peroxisome, and endoplasmic reticulum (ER) [23]. Considering that excessive ROS attack polyunsaturated fatty acids in biological membranes to trigger lipid peroxidation and produce a variety of oxidation products, causing cellular damage [24], the regulation of intracellular ROS levels is essential for intracellular homeostasis. MDA is a product of lipid peroxidation which can be used as indicators of oxidative stress [25].

The lipopolysaccharide (LPS) cell wall component of ETEC, a Gram-negative bacterium, is the main bacterial pathogenic factor [26]. Previously, it had been shown that IPEC-J2 cells treated with LPS can produce a large amount of MDA [27–29]. In this study, we show that ETEC induces the production of MDA in IPEC-J2 cells and that MDA content increases with increasing ETEC concentration. The highest MDA content and lowest cell viability recorded herein were achieved at the highest investigated concentration of ETEC K88 (1 × 10^3^ cfu/mL). In addition to bacterium concentration, the MDA content in the culture supernatant depends on the duration of the treatment. Indeed, the amount of MDA increases and cell viability decreases at longer incubation times. This suggests that ETEC may damage and destroy the cell membrane of IPEC-J2 cells. Compared to other fully differentiated organs, the cell in the intestinal is more susceptible to oxidative stress due to continuous exposure to exogenous substances [30]. Moreover, the fimbriae and adhesins of ETEC can strongly attach to intestinal cells, which promotes the long-term colonization and propagation of the bacteria in the intestine. The enterotoxin released by ETEC combines with the receptor of intestinal epithelial cells, thereby inducing an osmotic imbalance that impairs the function of the intestinal barrier and increases permeability. This indirectly leads to diarrhea and causes oxidative stress reaction, as reported in previous studies conducted on weaned piglets [31].

Under normal and healthy cell conditions (i.e., no oxidative stress), Nrf2 is anchored to the cytoskeleton of actin in the form of Keap1-Nrf2 complex. In the cytoplasm, the complex is inactivated by ubiquitination in order to maintain its low content and stable state [13]. However, when cells are subjected to oxidative stress, the cysteine residues at the active site of Keap1 are oxidized or covalently modified, resulting in the release of Nrf2 from Keap1. The activated Nrf2 is transported to the nucleus, where it binds to the Maf protein to form a heterodimer. Subsequently, the heterodimer combines with ARE, which activates the expression of a variety of downstream antioxidant genes and exerts antioxidant damage [32]. According to previous studies, LPS can significantly reduce the contents of Nrf2 and SOD in IPEC-J2 cells [27]. In addition, it induces apoptosis and decreases the expression of *GPX* in these cells [28]. Herein, we demonstrate that ETEC treatment decreases the contents of SOD and GPX in the supernatant of the IPEC-J2 cell culture. Moreover, lower contents are detected at higher concentrations of the bacterium (same treatment time). The lowest SOD and GPX contents reported in this study were measured at the ETEC K88 concentration of 1 × 10^3^ cfu/mL. Similarly, the amounts of SOD and GPX in the cell supernatant decrease at longer incubation times. Compared to the control group, the gene expression levels of *Nrf2*, *SOD1*, *SOD2*, *GPX1*, and *GPX2* in cells treated with 1 × 10^3^ cfu/mL ETEC K88 for 6 h are significantly decreased. Therefore, it may be concluded that at the concentration of 1 × 10^3^ cfu/mL, ETEC induces oxidative stress in IPEC-J2 cells by reducing the contents of antioxidant enzymes.

The hydrogen atom is a metabolite of CB that can enhance the activity of antioxidant enzymes, reduce oxidative stress, and improve the antioxidant capacity of the body by combining with free radicals [33]. Previous studies confirm that CB has many beneficial effects on animals, including oxidative stress reduction [34], intestinal flora balancing [35], lipid metabolism regulation [36], and the improvement of neurological deficit [37]. Pretreatment with 5 × 10^8^ cfu/mL CB decreases the content of MDA in the serum of mice suffering from gastric ulcer and improves the activities of SOD and CAT in gastric tissue [38]. Moreover, pretreatment with the probiotic alleviates the effect of CCl_4_-induced acute liver injury in increasing the level of MDA in mouse livers. At the same time, the pretreatment increases the activity of SOD and CAT in liver tissues, as well as the content of Nrf2 [12, 34]. The results obtained herein demonstrate that the expression levels of *Nrf2*, *SOD1*, *SOD2*, *GPX1*, and *GPX2* genes in IPEC-J2 cell pretreatment with CB followed by 12 h treatment with ETEC are significantly higher than those in cells treated with ETEC only. The level of Nrf2 protein in the CB+ETEC group is also higher. Previously, it had been shown that Nrf2 knockout in mice can lead to tumorigenesis and aggravating obesity due to oxidative stress [39, 40]. To assess the effect of *Nrf2* silencing on IPEC-J2 cells, we used siRNA to interfere with *Nrf2* gene expression. The obtained results indicate that the interferant significantly decreases the expression levels of *Nrf2*, *SOD1*, *SOD2*, *GPX1*, and *GPX2* in the siRNA and siRNA+ETEC groups compared to the control group. The levels of Nrf2 protein in the NC+ETEC and siRNA+ETEC groups are substantially lower than that detected in the control group. Moreover, the levels of these genes are lower in the siRNA+ETEC group than in the NC+ETEC group. These results indicate that the oxidative damage induced by ETEC is related to reduced *Nrf2* gene expression. After treatment with ETEC for 12 h and 18 h, the cells pretreated with CB (the NC+CB+ETEC and siRNA+CB+ETEC groups) exhibit significantly increased expressions of *Nrf2*, *SOD1*, *SOD2*, *GPX1*, and *GPX2* compared to the cells in the NC+CB+ETEC and siRNA+CB+ETEC groups. Also, the expression level of *Nrf2* in the siRNA+CB+ETEC group cells is lower than that in NC+CB+ETEC cells, which suggests that the probiotic effect of CB is decreased after transfection. Overall, the results obtained in this study indicate that CB protects IPEC-J2 cells from oxidative stress by promoting the activity of the Nrf2-mediated antioxidant enzyme system.

## 5. Conclusion

In general, the results reported in this study show that CB can inhibit the growth of ETEC K88, reduce its pathogenicity, and alleviate ETEC K88-induced oxidative damage by regulating the expression of key proteins implicated in the Nrf2/ARE signaling pathway. This information provides a theoretical basis for the application of CB in piglet diets.

## Figures and Tables

**Table 1:** Primer sequence information.

Target	Sequence/(5′ →3′)	Size (bp)	TM (°C)
*SOD1*	F:GAGACCTGGGCAATGTGACT	139	56
	R:CTGCCCAAGTCATCTGGTTT		
*SOD2*	F:TGGAGGCCACATCAATCATA	113	62
	R:TTTCGAAGGAACCAAAGTCG		
*GPX1*	F:TGAATGGCGCAAATGCTCAC	232	56
	R:GCTTCGATGTCAGGCTCGAT		
*GPX2*	F:TTGCCAAGTCCTTCTACGA	188	62
	R:GAAGCCAAGAACCACCAG		
*Nrf2*	F:CACCACCTCAGGGTAATA	125	56
	R:GCGGCTTGAATGTTTGTC		
*GAPDH*	F:GAAGGTCGGAGTGAACGGAT	149	62
	R:CATGGGTAGAATCATACTGGAACA		

## Data Availability

The data in this article support the findings of this study.
